# New Aspects of the Epigenetics of Pancreatic Carcinogenesis

**DOI:** 10.3390/epigenomes4030018

**Published:** 2020-08-31

**Authors:** Murat Toruner, Martin E. Fernandez-Zapico, Christopher L. Pin

**Affiliations:** 1 Schulze Center for Novel Therapeutics, Division of Oncology Research, Department of Oncology, Mayo Clinic, Rochester, MN 55905, USA;; 2 Department of Gastroenterology, Ankara University School of Medicine, School of Medicine, 06100 Ankara, Turkey; 3 Departments of Paediatrics, Oncology, and Physiology and Pharmacology, University of Western Ontario, Children’s Health Research Institute, London, ON N6C 2V5, Canada

## Abstract

Pancreatic cancer remains among the deadliest forms of cancer with a 5 year survival rate less than 10%. With increasing numbers being observed, there is an urgent need to elucidate the pathogenesis of pancreatic cancer. While both contribute to disease progression, neither genetic nor environmental factors completely explain susceptibility or pathogenesis. Defining the links between genetic and environmental events represents an opportunity to understand the pathogenesis of pancreatic cancer. Epigenetics, the study of mitotically heritable changes in genome function without a change in nucleotide sequence, is an emerging field of research in pancreatic cancer. The main epigenetic mechanisms include DNA methylation, histone modifications and RNA interference, all of which are altered by changes to the environment. Epigenetic mechanisms are being investigated to clarify the underlying pathogenesis of pancreatic cancer including an increasing number of studies examining the role as possible diagnostic and prognostic biomarkers. These mechanisms also provide targets for promising new therapeutic approaches for this devastating malignancy.

Pancreatic cancer is among the most aggressive gastrointestinal cancers, difficult to treat either by surgical intervention or conventional chemotherapy. Therefore, there is clearly a need to clarify the underlying mechanisms and to develop novel therapies based on these mechanisms. In 2000, a model for pancreatic cancer was presented based solely on genetic alterations [1]. In this model, pancreatic cancer arises from multiple genetic alterations, starting with mutations that promote the constitutive activation of KRAS (Kirsten Rat Sarcoma). However, it is now clear that mechanisms based purely on genetic mutations do not reveal the whole story. For example, pancreatic cancers with identical genetic alterations do not respond in a similar way to chemotherapy or show similar disease course.

Following the complete sequencing of the human genome in 2001 [2,3], our understanding of disease pathogenesis significantly improved. Pathogenesis for most neoplastic or non-neoplastic disorders cannot be solely explained by genetics or environmental factors. They both contribute to susceptibility, initiation and progression of most diseases. In pancreatic cancer, a significant gap in understanding the underlying processes regulating disease pathogenesis still exists. In this regard, epigenetic mechanisms have emerged as key mechanisms that could address this gap. The term epigenetics was originally described in the 1950s by Conrad Waddington to delineate changes in phenotype with no change in genotype [4,5]. In the current view, epigenetics is the study of mitotically heritable changes in the genome function without a change in the nucleotide sequence [6]. Recently, epigenetic mechanisms have emerged as possible explanations for the differential individual behavior of pancreatic cancers. This Special Issue focuses on the role of epigenetics in pancreatic cancer which is projected to be the second cause of cancer death by 2030.

The main epigenetic mechanisms can be grouped into three main categories—DNA methylation, modifications to core histone proteins comprising the nucleosome and RNA interference. By definition, epigenetic modifications are heritable and reversible through cell division [7]. In development, epigenetic modifications promote multipotent cells to differentiate into more defined cell types, with new phenotypes transferred to daughter cells [8]. DNA methylation is established by DNA methyltransferases (DNMTs), which include the maintenance DNMT1 and the de novo methyltransferases DNMT3a/b and DNMT3L [9]. Conversely, DNA demethylation can occur through both passive demethylation through inhibition of methylation and active enzymatic-based demethylation.

Histone modifications occur mainly in the tail regions of the histones, and include acetylation, methylation, phosphorylation, and ubiquitination amongst other modifications. Post-transcriptional modifications to histone structures are usually kept in balance [10]. When the balance is distorted in either way, it leads to gene overexpression or gene repression. Finally, RNA interference refers to noncoding RNAs such as microRNAs (miRNAs) and long noncoding RNAs (lncRNAs), which modulate gene expression in a sequence specific fashion [11,12].

In this issue, recent advances in epigenetic mechanisms that control pancreatic carcinogenesis and the potential for targeting these mechanisms for diagnostic or therapeutic purposes will be discussed. This issue also includes reviews on the role of the nuclear structure and nutriepigenomics in pancreatic cancer. In each article, authors provide insight regarding potential therapeutic opportunities in targeting epigenetic mechanisms.

There are two main precursor lesions resulting in pancreatic ductal adenocarcinoma (PDAC) formation—pancreatic intraepithelial neoplasia (PanIN) and intraductal papillary mucinous neoplasm (IPMN). From a genetic mutation perspective, more than 90% of pancreatic cancer patients have gain of function *KRAS* mutations, and tumor suppressor genes such as *SMAD4* and *p53*, are frequently inactivated. However, no clear correlation has been established between the genetic subtypes of pancreatic cancer and these lesion types, nor the transcriptional subtypes more recently identified including squamous, pancreatic progenitor, exocrine-like (ADEX) and immunogenic PDACs. This lack of correlation highlights possible underlying epigenetic mechanisms. Hank and Liss review these basic epigenetics mechanisms in pancreatic cancer. They summarize basic epigenetic mechanisms that pertain to pancreatic cancer, including histone acetylation and deacetylation models, histone methylation and bromodomain and extraterminal (BET) regulation. Recent advances in chromatin regulation and the potential role of such regulation in PDAC pathogenesis are highlighted.

Cellular function is affected by chromatin control through several mechanisms, including structural mechanisms. Disruption of the nuclear structure can have significant consequences that contribute to oncogenesis. Preston and Faustino describe how the nuclear envelope plays a key role in controlling chromatin processes that are crucial for regulating PDAC pathogenesis. They spotlight three specific components of the nuclear envelope including the LINC (linker of nucleoskeleton and cytoskeleton) complex, which structurally couples cytoskeleton to chromatin and facilitates gene regulation, the nuclear lamina, which provides structural integrity to nucleus, and the nuclear pore complex, which mediates bidirectional nucleocytoplasmic transport. In addition, they describe how components of the nuclear pore complex—nucleoporins—are used as diagnostic markers in cancer.

While mortality rates of cancer are generally declining, pancreatic cancer is a notable exception, likely due to late diagnosis and resistance to conventional treatments. Epigenetic mechanisms are likely to contribute to these factors in pancreatic cancer. Hamdan and Johnsen review epigenetic targets as potential treatment options for pancreatic cancer. They highlight inhibitors for BET proteins and histone deacetylases (HDAC) that exert direct or synergistic effects with other chemotherapeutic agents. They emphasize how mediating their effects on distal regulatory elements, BET and HDAC inhibitors may be effective in PDAC treatment.

In our current understanding of PDAC progression, there are two proposed models. The first involves clonal evolution and suggests tumors are biologically homogenous, derived from the same clone. The second model includes stem cells in which heterogeneity exists across tumor cells providing a defined hierarchical structure within the tumor. Recent studies suggest both models contribute to tumor heterogeneity. Zagorac, Garcia-Bermejo and Sainz describe the epigenetic landscape of cancer stem cells. They provide a summary of the effects that DNA methylation, histone modifications and miRNAs have on cancer stem cells. Moreover, they outline possible epigenetic therapies that target these mechanisms in cancer stem cells.

There is a significant body of research on the association between nutrition and pancreatic cancer. The study of nutrients and their effects on human health through epigenetic mechanisms is termed Nutriepigenomics. However, how specific diets increase or decrease cancer risk is still unclear, as well as the effects that diet has on underlying epigenetic mechanisms. Specific to PDAC, epidemiological studies show folate levels, fruit/vegetable consumption, red meat consumption and saturated fat can alter pancreatic carcinogenesis. Epigenetic mechanisms linked to diet include folate changes that induce donation of methyl groups, and diallyl disulfide and sulforaphone inhibition of histone deacetylase [13,14], which stimulate apoptosis and inhibit cell proliferation.

Understanding epigenetic dysregulation mechanisms will contribute to our efforts in early diagnostic biomarker development, define predictive and prognostic biomarkers and identify new therapeutic approaches in pancreatic cancer. Thompson and Bednar review the clinical aspects of epigenetic changes in PDAC. They describe how genome wide assessment of changes in methylation status gave rise to next generation diagnostic assays. Cohen et al. describe a parallel approach for the testing of protein markers and circulating free DNA (cfDNA) with high specificity [15]. Epigenetic changes, including *ARID1A*, *MLL1*, *MLL2*, and *MLL3* mutations or altered DNA methyltransferase 1 expression, alter survival in patients with pancreatic cancer [16,17]. Thompson and Bednar also describe the relationship between epigenetic mechanisms and pancreatic cancer diagnosis, prognosis and survival.

In total, this issue makes a strong case for future studies and a better understanding of the epigenetic mechanisms that regulate pancreatic cancer initiation and progression. Through continued work in these areas, better prognostic and diagnostic markers will be identified, patient-specific treatment options will be supported and novel combinations of epigenetic and nonepigenetic drugs will be investigated.
